# The decades-old mystery of bis­(diethyl ether)tungsten(IV) chloride solved

**DOI:** 10.1107/S2053229621002461

**Published:** 2021-03-12

**Authors:** André Schäfer

**Affiliations:** a Department of Chemistry, Saarland University, Campus Saarbrücken, Saarbrücken, Saarland 66123, Germany

**Keywords:** tungsten, diethyl ether complex, octa­hedral, coordination chemistry, commentary

## Abstract

Several decades after the bis­(diethyl etherate) of tungsten(IV) chloride was first mentioned in the literature, it has now at last been structurally characterized by single-crystal X-ray diffraction analysis, finally confirming its composition.

A hexa­coordinated metal centre with an octa­hedral coordination environment is a very common motif in transition metal chemistry. This can involve neutral, as well as anionic, ligands or a combination of both. The latter can, *inter alia*, be found in many Group 6 complexes, often with donor solvent mol­ecules acting as neutral ligands and saturating the coordination sphere of the metal(IV) centre. In particular, with regard to tungsten, many complexes of the type W*X*_4_(solvent)_2_ are known in the literature and are even found in textbooks nowadays, as, for instance, WCl_4_(py)_2_ (Brenčič *et al.*, 1979 ▸) or WCl_4_(MeCN)_2_ (Manteghetti *et al.*, 1999 ▸), which have even been structurally characterized by single-crystal X-ray diffraction. A common, yet ambiguous, example of this is bis­(diethyl ether)tungsten(IV) chloride, WCl_4_(Et_2_O)_2_. Although this compound has been ‘floating around’ in the literature for many decades, surprisingly little analytical data concerning it have been reported, begging the question if it even truly exists.

Taking a look at the history of the compound, it starts in 1975 when Thiele *et al.* (1975 ▸) studied different benzyl­tungsten compounds, tetra­benzyl­tungsten amongst others, which they obtained from the reaction of di­benzyl­magnesium with tungsten(IV) chloride bis­(tetra­hydro­furan), WCl_4_(thf)_2_. This, in turn, was synthesized from tungsten(VI) chloride, WCl_6_, by treatment with di­ethyl­zinc in diethyl ether, whereas the authors proposed that the tungsten(IV) chloride bis­(diethyl ether) adduct, WCl_4_(Et_2_O)_2_, was formed initially, and was then transformed into the isolatable bis­(tetra­hydro­furan) adduct. Unfortunately, no analytical data for the bis­(diethyl ether) complex was provided. At roughly the same time, Chisholm, Cotton and co-workers investigated the use of WCl_4_(Et_2_O)_2_ as a possible precursor in the preparation of tungsten–tungsten triple-bond complexes (Chisholm *et al.*, 1976*a* ▸,*b* ▸). While WCl_4_(Et_2_O)_2_ was explicitly mentioned as a bis­(diethyl etherate), it is worth pointing out that it was prepared *in situ* (by the same route as described above), with a 100% yield assumed, and was used without characterization. Therefore, although WCl_4_(Et_2_O)_*x*_ had already proven to be an inter­esting synthon, the exact nature and structure of the complex remained unclear and its composition, suggested to be a bis­(diethyl ether) adduct, was based simply upon chemical intuition and the logical assumption of an octa­hedral complex with hexa­coordinated tungsten (Fig. 1 ▸).

Almost a decade later, in 1985, Castellani & Gallazzi (1985 ▸) revisited the preparation of diethyl ether complexes of molybdenum and tungsten tetra­chlorides, and made an attempt to characterize WCl_4_(Et_2_O)_*x*_ by elemental analysis to elucidate its exact composition. In contrast to previous articles, they reported that the mono(diethyl etherate), WCl_4_(Et_2_O), was obtained from diethyl ether solutions of WCl_6_, which was based on elemental analysis suggesting not two but one equivalent of C_4_H_10_O [WCl_4_(Et_2_O)_2_: C 20.3, H 4.3%; WCl_4_(Et_2_O): C 12.0, H 2.5%; reported by Castellani & Gallazzi (1985 ▸): C 11.5, H 2.4%]. First, it is worth mentioning that Castellani & Gallazzi seem to be the first to actually attempt to isolate the complex as a pure solid, rather than just synthesize and use it *in situ*. Furthermore, they reported that an orange reaction mixture, along with a yellow solid, formed initially, but that ultimately black crystalline needles were obtained and isolated. It seems odd that a compound could be obtained in the form of black crystals from an orange reaction mixture and, taking into account that a yellow precipitate was observed initially, this might point towards an equilibrium between a mono- and bis­(diethyl etherate), due to the relatively weak complexation of the diethyl ether ligands. In fact, Castellani & Gallazzi pointed out correctly that other complexes, such as WCl_4_(thf)_2_ and WCl_4_(py)_2_, always feature two complexed solvent mol­ecules.

It is puzzling that for decades this mystery remained unsolved, even though this complex was frequently used as a starting material in tungsten chemistry. Finally, in 2021, almost half a century after the complex first appeared in the literature, Jurca and co-workers were able to obtain a pure crystalline sample of the bis­(diethyl ether) complex, WCl_4_(Et_2_O)_2_, and conduct a complete structural characterization by single-crystal X-ray diffraction and NMR spectroscopy (Shaw *et al.*, 2021 ▸). Different from previous reports, the authors used tungsten tetra­chloride, WCl_4_, as the starting material, dissolving it in diethyl ether. In a striking similarity to the report of Castellani & Gallazzi from 1985, they observed a yellow solution and were able to obtain not a black but a yellow crystal from it. Structural characterization of this crystal by X-ray diffraction confirmed it to be tungsten tetra­chloride bis­(diethyl etherate), WCl_4_(Et_2_O)_2_, with *trans*-coordinated diethyl ether mol­ecules in the axial positions (Fig. 1 ▸)! With this, the quest for the elucidation and structural characterization of the long-discussed WCl_4_(Et_2_O)_2_ finally comes to an end, closing this gap in tungsten tetra­chloride bis­(solvent) complexes.

The question remains, did Castellani & Gallazzi actually obtain the mono(diethyl etherate), WCl_4_(Et_2_O), in 1985, as suggested? It seems not unlikely given the fact that diethyl ether is a rather weakly bonded ligand, and they reported black crystals instead of yellow ones and the elemental analysis was very much in line with a mono(diethyl etherate). Two inter­esting details are given by Jurca and co-workers here as well, namely, that ‘prolonged exposure to ambient conditions led to the appearance of dark spots on the crystal surface’ and that ‘the compound is unstable under vacuum’ (Shaw *et al.*, 2021 ▸). This clearly suggests the loss of one equivalent of diethyl ether due to rather weak complexation, which would explain Castellani & Gallazzi’s elemental analysis results.

## Figures and Tables

**Figure 1:**
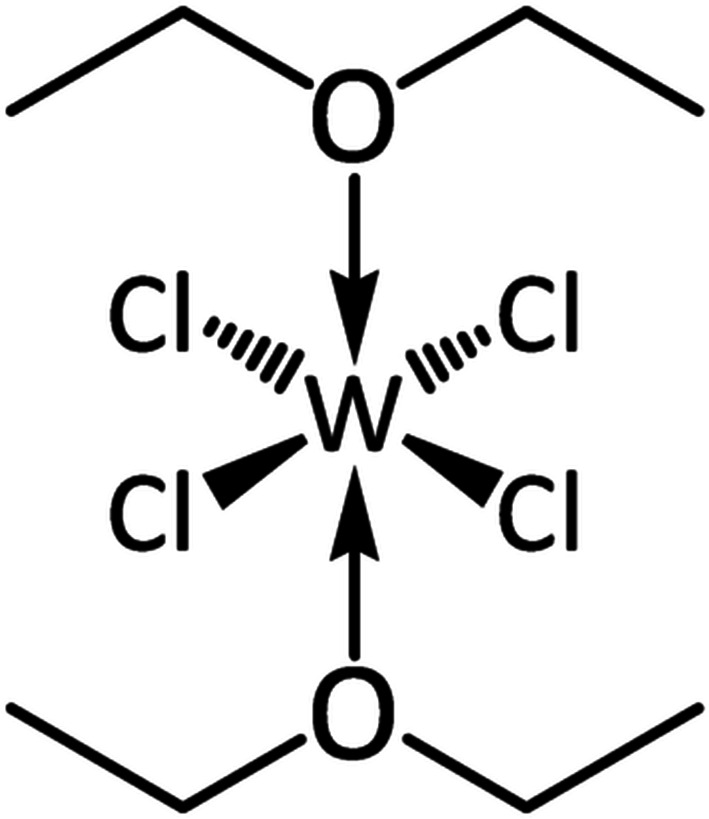
Tungsten tetra­chloride bis­(di­ethyl etherate), WCl_4_(Et_2_O)_2_.
